# Microstructure and Properties of Mg-Gd-Y-Zn-Mn High-Strength Alloy Welded by Friction Stir Welding

**DOI:** 10.3390/ma17174190

**Published:** 2024-08-24

**Authors:** Jinxing Wang, Zhicheng Wan, Xiyu Wang, Jiaxu Wang, Yi Zou, Jingfeng Wang, Fusheng Pan

**Affiliations:** 1 College of Materials Science and Engineering, Chongqing University, Chongqing 400030, China; 202209021059@stu.cqu.edu.cn (Z.W.); 17725026348@163.com (X.W.); 202309131157@stu.cqu.edu.cn (J.W.); 202209021040@stu.cqu.edu.cn (Y.Z.); fspan@cqu.edu.cn (F.P.); 2 National Engineering Research Center for Magnesium Alloys, Chongqing University, Chongqing 400030, China

**Keywords:** magnesium alloys, MVWZ842 alloy, friction stir welding, mechanical properties, strengthening mechanisms

## Abstract

Mg-Gd-Y-Zn-Mn (MVWZ842) is a kind of high rare earth magnesium alloy with high strength, high toughness and multi-scale strengthening mechanisms. After heat treatment, the maximum tensile strength of MVWZ842 alloy is more than 550 MPa, and the elongation is more than 5%. Because of its great mechanical properties, MVWZ842 has broad application potential in aerospace and rail transit. However, the addition of high rare earth elements makes the deformation resistance of MVWZ842 alloy increase to some extent. This leads to the difficulty of direct plastic processing forming and large structural part shaping. Friction stir welding (FSW) is a convenient fast solid-state joining technology. When FSW is used to weld MVWZ842 alloy, small workpieces can be joined into a large one to avoid the problem that large workpieces are difficult to form. In this work, a high-quality joint of MVWZ842 alloy was achieved by FSW. The microstructure and properties of this high-strength magnesium alloy after friction stir welding were studied. There was a prominent onion ring characteristic in the nugget zone. After the base was welded, the stacking fault structure precipitated in the grain. There were a lot of broken long period stacking order (LPSO) phases on the retreating side of the nugget zone, which brought the effect of precipitation strengthening. Nano-α-Mn and the broken second phase dispersed in the matrix in the nugget zone, which made the grains refine. A relatively complete dynamic recrystallization occurred in the nugget zone, and the grains were refined. The welding coefficient of the welded joint exceeded 95%, and the hardness of the weld nugget zone was higher than that of the base. There were a series of strengthening mechanisms in the joint, mainly fine grain strengthening, second phase strengthening and solid solution strengthening.

## 1. Introduction

There are many advantages to magnesium alloy, such as lightweight, high strength, good processability, and excellent electromagnetic shielding, etc., [1,2,3]. It is internationally recognized as a lightweight material with great structural and functional integration potential. The addition of rare earth can lead to multiple strengthening mechanisms [4,5,6], so that the mechanical properties and the service range of magnesium alloys can be significantly improved [7,8]. High-strength and high-toughness magnesium alloys have become key materials for promoting industrial lightweighting and improving product performance due to their unique properties. The addition of high rare earth increases the deformation resistance of magnesium alloy, and it is challenging to realize the formation of large structural parts [9]. By studying high-quality connection processes and applying them to rare earth magnesium alloys, it is possible to assemble small workpieces into large ones. It can avoid the forming difficulty of large-scale complex structural parts of rare earth magnesium alloy and accelerate the industrial application [10]. In the early stage of this work, a high-strength MVWZ842 alloy with multi-scale strengthening mechanisms was obtained by co-regulating the morphology of the LPSO phase and the distribution of nano-precipitated phases γ’ and β’. The strengthening mechanism mainly includes three aspects. (1) The LPSO phase itself is a high-modulus and high-hardness phase, with a good second phase strengthening effect. The block-shaped LPSO after hot processing exhibits fiber distribution with a composite phase strengthening effect. (2) The LPSO phase can undergo twisting at any angle to promote uniform deformation and suppress twinning because of good plasticity [11]. Twisting generates a large number of dislocations. Then, a dislocation wall will form and further enhance the strength. (3) The LPSO phase also has a grain refinement effect during hot deformation [12]. While the maximum tensile strength exceeds 555 MPa, the elongation of MVWZ842 exceeds 5% [13,14,15]. Due to their excellent properties, rare earth magnesium alloys with high strength and high toughness possess broad application prospects in the aerospace, rail transit and car industry fields [16,17,18].

However, there are few slip systems in magnesium alloys because of the hexagonal structure. The plasticity of magnesium alloys is limited at room temperature [19]. To avoid direct plastic forming of large workpieces, there is an urgent need for connection technology suitable for rare earth magnesium alloys. Friction stir welding, as a solid-state joining technology, was invented by the Welding Institute in 1991 [20]. It can realize joining by mixing large plastic deformation and material flow in the solid state, effectively avoiding the shortcomings of fusion welding [21,22,23]. It has been widely applied in aviation, rail transit and the automotive industry [10,24]. At present, many studies have shown that friction stir welding is a reliable means to realize the effective connection of Mg-RE alloys [25,26]. The use of friction stir welding to prepare high-strength and large-size rare earth magnesium alloy profiles is beneficial to broaden the application field of rare earth magnesium alloy [25,27].

At present, there are relatively few reports about using FSW to connect MVWZ842 alloy with high-quality products [24]. The FSW process for this alloy is lacking and the relationship between the process and the formability is not clear. In this work, FSW was selected to study the welding of MVWZ842 alloy. The microstructure and properties of the joint were characterized, and its strengthening mechanism was analyzed. This study is expected to help explore the welding process specification of this alloy and lay a foundation for its engineering application.

## 2. Materials and Methods

### 2.1. Materials

The base used for welding is Mg-Gd-Y-Zn-Mn alloy, which has not been overheated and developed in the early stage of this study. Its practical chemical composition is shown in Table 1. The initial state of the alloy is a hot extruded profile, and the strength of the base alloy is shown in Table 2.

### 2.2. Welding Experimental Procedure

The experiment uses LT-XJ16-1510-05 static gantry friction stir welding equipment (Liutai Welding Technology Co., Ltd., Guangdong, China). The welding method is butt joint, and both the retreating side (RS) and the advancing side (AS) are made of MVWZ842 alloys. The dimensions of the board are 150 mm ×70 mm ×3.8 mm. The length direction of the sheet is the extrusion direction (ED), the width direction is the transverse direction (TD) and the thickness direction is the normal direction (ND). A stir-welding head consisting of a concave shoulder with a diameter of 14 mm and a conical welding pin with a right thread is selected. During welding, the inclination angle of the stir-welding head is fixed at 2.5°, the pressing speed is fixed at 20 mm/min and the dwell time is fixed at 3 s. The welding experiment process is divided into the following three steps: first, the sheet’s surface is polished and cleaned, then the magnesium alloy sheet is fixed and, finally, the friction stir welding begins. After welding is completed, a wire cutting method is used to cut a metallographic sample of the cross-section of the joint at the weld nugget. The sample size is 20 mm (TD) ×3.8 mm (ND) ×4 mm (welding direction, WD), and the observation surface is ND × TD.

### 2.3. Microstructural Observations

The metallographic samples are corroded after polishing, and then the microstructure is observed under OLYMPUS OLS4000 metallographic microscope (Juna Technology Co., Ltd., Shanghai, China). Field emission scanning electron microscopy (SEM) JEOL JSM-7800F (Japan Electronics Co., LTD, Tokyo, Japan) is used to observe the welded joints. The morphology characteristics of each region of the welded joint are observed by secondary electron imaging, the morphology and distribution characteristics of the second phase are observed by backscattered electron imaging mode (BED), the orientation texture of the welded joint is analyzed by electron backscattering diffraction (EBSD) probe and the composition of the second phase is analyzed by energy dispersive spectroscopy (EDS). X-ray diffraction (XRD) phase analysis of the base alloy and the welded joint is carried out by an assembly X-ray diffractometer (Rigagu, Tokyo, Japan). Field emission transmission electron microscopy (TEM) Tecnai G2 F20 (FEI Company, Hillsboro, OR, USA) is used to characterize the weld nugget zone.

### 2.4. Mechanical Properties Test

An HXS-1000ZY microhardness tester (Minsk Instrument Co., Ltd., Shanghai, China) is used to conduct microhardness testing on various areas of the welded joint. For microhardness specimens, the measuring surface is also the ND-TD surface, with dimensions of 40 mm (TD) ×3.8 mm (ND) ×5 mm (WD). Before testing, the test surface is subjected to metallographic water grinding to a 1200 mesh. During testing, dots are placed along the TD direction at intervals of 0.5 mm, with a load of 200 g and a holding time of 10 s. The room temperature tensile test is conducted on the CMT-5105 electronic universal material testing machine, with the loading direction parallel to the specimen axis and a tensile rate of 1 mm/min. The yield strength is recorded as the alloy strength at the specified non-proportional extension (0.2%). The tensile specimen is a sheet-like specimen, cut along the TD of the plate to ensure that the weld nugget zone is located at the center of the tensile specimen. The total length of the stretched sample is 78 mm, the gauge length is 34 mm and the thickness is 3.8 mm.

## 3. Results

### 3.1. The Structure of the Joint

**Figure 1:**
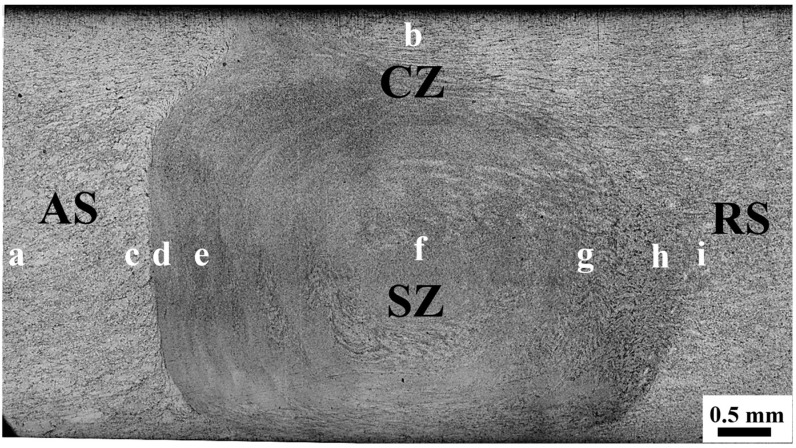
Macroscopic feature of the joint.

The macro-morphology of the weld cross-section is shown in Figure 1.(The areas a-i are the regions for subsequent magnification observation) The whole weld is bowl-shaped, and the weld nugget comprises a crown zone (CZ) and a stir zone (SZ). Both sides of the material flow mixing are sufficient, with no holes, cracks or other macro-defects. The direction of material flow on the forward side is opposite to the direction of the stirring needle rotation, the strain shear effect is large and the interface is the clearest. The material flow direction of the backward edge is the same as that of the mixing head, so the interface is relatively indistinct. In addition, black-and-white features are similar to onion rings in the weld nugget zone [28]. Xin et al. [29] conducted FSW on Mg-Gd-Y-Nd-Zr in both solid solution and aged states, and found significant onion rings in the aged alloy. Through in-depth characterization, it was found that onion rings are composed of alternating banded structures with different grain sizes and phases. Some scholars believe that this layered stacking structure with different structures and properties can play a role in the effect of layered composite strengthening, which can simultaneously improve strength and plasticity [30].

**Figure 2:**
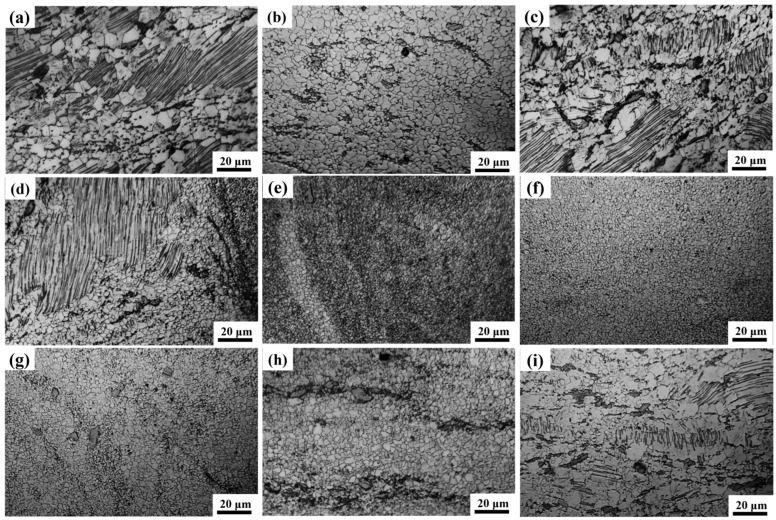
Metallographic structure of each area of the joint. (**a**) Base; (**b**) crown zone (**c,d**) transition zone on the AS; (**e**–**g**) stirring zone; (**h**,**i**) transition zone on the RS.

To analyze the microstructure of each region more clearly, the base, the transition zone on the AS, the nugget zone, the transition zone on the RS and the crown zone are characterized. These areas correspond to the positions a–i in Figure 1 in turn. The metallographic structure of the base is shown in Figure 2a, and it can be seen that the structure of the base has the characteristics of a bimodal structure. It consists of recrystallized equiaxed grains and un-recrystallized large deformed grains with an average grain size of 7.7 μm. It is worth noting that layered LPSO phases in large grains have not been recrystallized. This may be because the layered LPSO phase is prone to twist and release strain energy during thermal deformation, which inhibits recrystallization. So a large number of deformed grains containing layered LPSOs are retained [15]. Figure 2b shows the structure of the crown zone. Complete dynamic recrystallization occurred in the crown zone. Because of the highest surface temperature of the weld, the grains in the crown zone are coarser than those in the stirring zone, with an average grain size of 3.9 μm. As shown in Figure 2c,d, in the transition zone on the AS, equiaxed grains and deformed grains partially recrystallize under the action of strain and high temperature. The grain boundary profile becomes zigzag and convex with the characteristics of discontinuous dynamic recrystallization. The newly generated recrystallized grains are the same as those in the nugget zone. The layered LPSO phase in the transition zone also bends and twists, and the blocky LPSO phase is streamlined along the edge of the nugget zone. Figure 2e–g show the microstructure of the stir zone from AS to RS, and the grain is refined to about 1 μm under intense stirring and shearing. There are a lot of particles in the AS of the stir zone, and the onion ring is a black-and-white banded structure, while the central matrix in the stir zone is relatively pure. Due to the relatively small strain on the RS of the stir zone, the second phase breaks into particles but is not uniformly dispersed but flocculent. In addition, there are also blocky LPSO phases with incomplete fragmentation. Figure 2h–i show the microstructure of the transition zone from the RS of the weld nugget to the base in turn. The microstructure of the weld nugget’s RS is in continuous transition with the base, so it is difficult to judge its interface. The grains in the transition zone are refined, and the size is slightly larger than that in the nugget zone. However, the second phase changes continuously, and the blocky LPSO phase is elongated and distributed in streamline under shear. The residual layered LPSO phase appears at a certain distance from the weld nugget, and the layered LPSO phase originally distributed across the grain dissolves and shortens under thermal deformation. Finally, because of the weakening of thermal action, an incomplete dynamic recrystallization structure with large and small grains appears. Because of the various temperature and heating effects, the discrepancy regions of the joint display different microstructures.

### 3.2. The Second Phase of Joint

**Figure 3:**
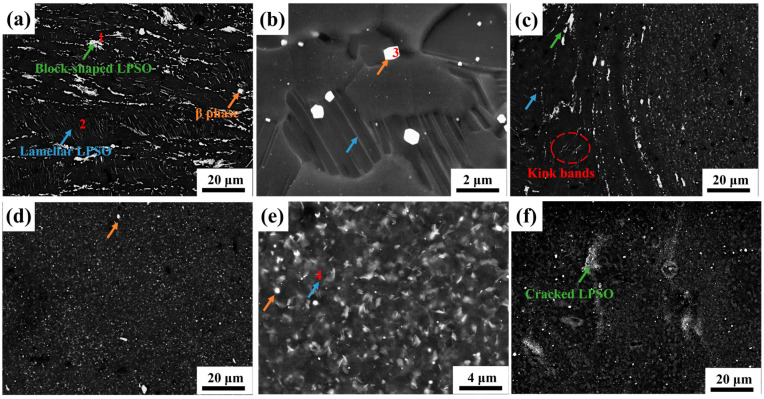
SEM image of FSW joint. (**a**) Base; (**b**) partial enlarged view of base; (**c**) advancing side transition zone; (**d**) nugget center; (**e**) enlarged partial view of weld nugget; (**f**) retreating side transition zone. (The red number represents the location of the EDS dot analysis).

To observe the second phase morphology of the joint more directly, the SEM image and EDS results of the FSW joint are shown in Figure 3 and Table 3, respectively. According to Figure 3a and EDS results, the base mainly comprises blocky LPSO, layered LPSO and a eutectic phase with bright contrast. Figure 3b is a locally enlarged image of the base, and EDS analysis shows that the eutectic phase is Mg_5_(RE). Because of a high melting point (658 ℃), some Mg_5_(RE) has been retained in the FSW process. As shown in Figure 3c, the AS interface of the joint is visible, the blocky LPSO is distributed along the interface flow line and the layered LPSO is dissolved by twisting. As shown in Figure 3d, the second phase in the nugget area is completely broken and evenly distributed in the matrix after undergoing high temperature and severe deformation. According to Figure 3e, dense layered LPSO is distributed in the grain in the weld nugget zone. These layered LPSO phases may be precipitated from the matrix or transformed from the blocky LPSO at high temperatures. Figure 3f shows the secondary electron figure of the RS of the weld nugget zone. Under the rotating shear force, the original blocky LPSO is broken into fine particles. However, because the material flow direction of the RS is the same as the rotation direction, the strain variable is relatively small, and the second phase is not completely dispersed. The morphology of LPSO will affect the joint performance. For example, the dispersion of LPSO usually refines the grain and strengthens the joint properties.

**Figure 4:**
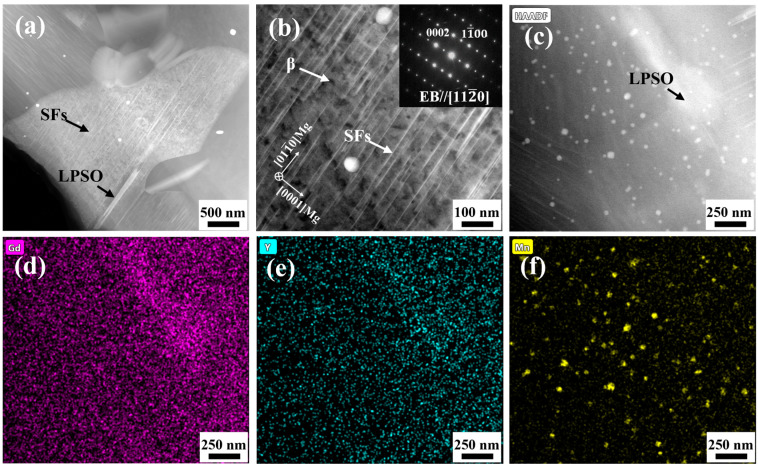
TEM microstructure of the joint. (**a**) HAADF-STEM; (**b**) HAADF-STEM and corresponding selected area electron diffraction patterns; (**c**) HAADF-STEM; (**d**–**f**) EDS surface scan results of figure.

**Figure 5:**
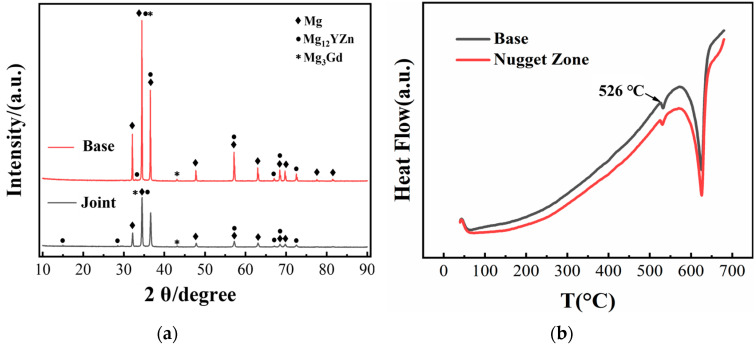
(**a**) XRD diagram of MVWZ842 alloy base and welded joint; (**b**) DSC curve of MVWZ842 alloy base and welded joint.

To further observe the microstructure of the joint, the TEM image of the welded joint and the EDS surface scan results of selected areas are shown in Figure 4. Figure 4a–c show a high-angle annular darkfield figure (HAADF) of the weld nugget region, and its contrast reflects the magnitude of the atomic number. As shown in Figure 4a, the blocky LPSOs distributed at the original grain boundaries can be broken into short slats under low-magnification HAADF. As can be seen in Figure 4b, the ultrafine grains are uniformly distributed with small parallel and dense flakes. They are identified as stacking faults (SFs) based on the selective electron diffraction pattern along the [11$\bar{2}$0] ribbon axis. The stacking faults in the grains maintain a coherent relationship with the Mg matrix, which proves that the stacking faults are precipitated in the grains. The EDS surface scanning is performed on the field of view area in Figure 4c. It can be found that there are Gd and Y atoms. The presence of Gd and Y atoms diminish the stacking fault energy of the α-Mg matrix. This may be the reason why the dislocations first nucleate and then the Schottky incomplete dislocation extends to the formation of the stacking faults. The structure should be an intermediate structure of 14H-LPSO. The fast welding speed and short duration of high temperature limit the further formation of the LPSO phase [31]. In addition, it is found that some nanoscale bright particles are distributed in the grain, and the particles are mainly α-Mn elemental by energy spectroscopy analysis, as shown in Figure 5e. The dispersed fine Mn elemental inhibits the growth of recrystallized grains during thermal deformation.

The XRD results of the MVWZ842 alloy base and welded joint are shown in Figure 5a. The XRD results show that the weld and base have the same angle diffraction peak, indicating that the phase composition of the two is the same. The DSC curves of the MVWZ842 alloy base and welded joints are shown in Figure 5b The DSC results show that the nugget and base have the same heat absorption peak at 527 ℃. This peak is due to the heat absorption of the LPSO phase decomposition. The high thermal stability of the LPSO phase makes the weld undergo high-temperature strain. The phase is not dissolved in large quantities but is broken and dispersed, which brings a significant precipitation-strengthening effect. This is consistent with the previous morphological observations.

### 3.3. The Texture of the Joint

**Figure 6:**
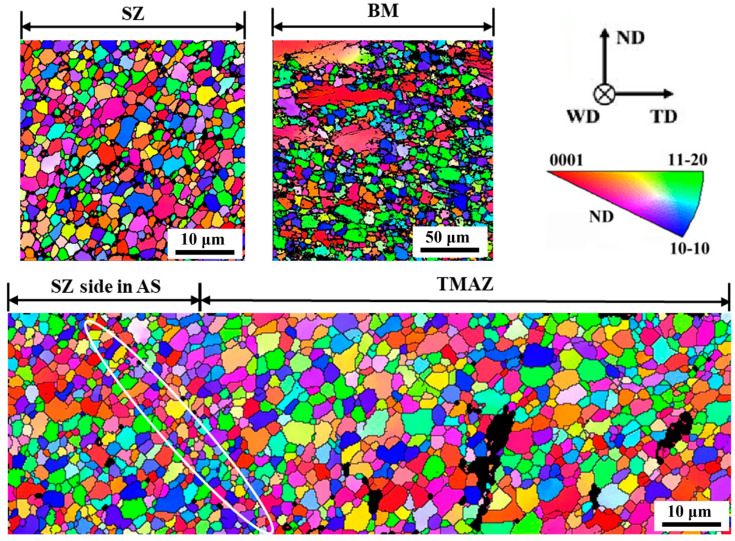
IPF map of the cross-section of the welded joint. (The white oval marks the edge of the stir zone).

The grain size and orientation of the welded joint will influence the performance of the joint. The inverse pole figure (IPF) for the joint cross-section is shown in Figure 6. The base is hot extruded and possesses obvious bimodal structure characteristics. It comprises large deformation grains without recrystallization. The fine recrystallization grains have an average grain size of 5.05 µm. The deformed grains show a strong basal texture, while the orientation of recrystallized grains is random. After high temperature and large plastic deformation, the grain orientation is randomly distributed. The grains are completely refined, with an average grain size of 1.47 µm. Recrystallization also occurs in the transition zone at the edge of the weld nugget zone to a large extent. The recrystallization grain size is slightly larger than that in the weld nugget zone (average grain size 2.77 µm) due to the weakening of thermal action in the transition zone. It is worth noting that there is also a fine crystal band at the edge of the stirring zone, which is mainly related to the high strain rate here.

**Figure 7:**
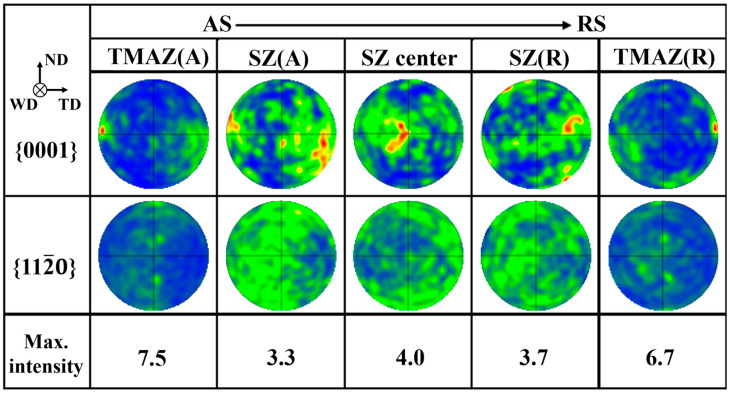
Pole diagrams of various regions in the welded joint.

To accurately analyze the texture of the welded joint, the different areas of the joint are observed. Figure 7 shows the {0001} and {11$\bar{2}$0} polar diagrams of different areas. The results show that the joint forms a welded texture similar to that of AZ series magnesium alloy after FSW. Under the action of rotating shear force, the c-axis of the grain is distributed in the normal direction towards the surface of the stirring needle. The c-axis of the thermo-mechanical affected zone (TMAZ) is roughly parallel to the TD, with a maximum texture strength of 7.5 times random texture. As the center of the stir zone approaches, the c-axis of the grain is inclined toward the weld direction (WD). In the center of the stir zone, the c-axis of the grain is almost parallel to the WD. The orientation distribution of the AS and the RS are the same. However, compared with the AZ strong texture after welding, the alloy texture of the weld nugget is weak, and the maximum texture strength is only 3.3–4 times of random texture. As for the reason for texture weakening, it is speculated that Gd and Y rare earth elements are related. Rare earth elements can affect the orientation distribution of deformation and dynamic recrystallization (DRX) process through the following three mechanisms: (1) The addition of a large number of Gd and Y atoms can reduce the stacking fault energy and the critical resolved shear stress (CRSS) of the non-base, promote <c+a> non-base slip and make the texture more disordered. (2) Rare earth atom segregation at the grain boundary changes the grain boundary energy and produces a strong solute atom dragging effect on dislocation and grain boundary. It affects the orientation of recrystallized grains and forms an abnormal rare earth texture (0001)//extrusion direction (ED). The rare earth texture-oriented grains grow preferentially, while the basic-oriented grains grow slowly and are absorbed by rare earth-oriented grains [32]. (3) When large second phase particles (no less than 0.1 µm) are thermally deformed, a strain zone is formed around the particles, which promotes recrystallization nucleation and randomization of orientation.

### 3.4. The Mechanical Properties of the Joint

**Figure 8:**
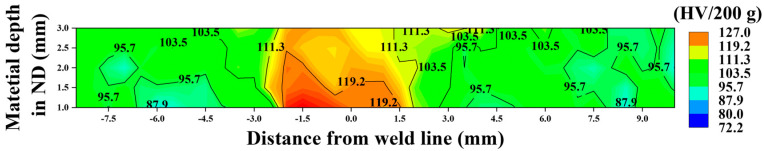
Hardness distribution of welded joint.

The hardness test is made in each area. The hardness distribution of the joint is depicted in Figure 8. It is observed from the figure that the hardness of the base is approximately 95 to 105 HV. The hardness of the weld nugget zone is significantly higher than that of the base, being around 110 to 123 HV. The hardness within the weld nugget zone is not uniformly distributed. The hardness of the stirred zone is higher than that of the crown zone, and the hardness at the AS of the weld nugget zone is also slightly higher than that at the center. The variations in hardness distribution within the weld nugget zone are related to the distribution of welding temperatures and the plastic flow of the materials. The crown zone, which is adjacent to the shoulder, experiences the highest peak temperature, resulting in relatively larger grains and lower average hardness in this area than the stir zone. The edge of the stir zone, due to the maximum strain rate, owns finer grains and greater hardness. In addition, comparing the microstructure between the center and the edge of the welding nugget zone, it can be found that the second phase in the center of the welding nugget zone has a high degree of solid solution. The matrix is relatively pure, and the fraction of the second phase in the edge is higher. These differences lead to the hardness of AS being higher than that of the weld nugget zone. The hardness variation on the RS is more continuous and gentle compared with the AS, which corresponds to the changes in the microstructure.

It is noteworthy that the hardness in all regions of the joint did not fall below that of the base, indicating that the welding process did not result in any regions of performance degradation. For some magnesium alloys dominated by precipitation strengthening, the joint hardness distribution shows a “V-shaped” pattern [33]. The hardness distribution in joints of non-rare earth magnesium alloys often exhibits a “W-shaped” pattern. It is attributed to the thermal cycle effects on the heat-affected zone (HAZ), leading to significant grain growth. For some magnesium alloys in which precipitation strengthening is predominant, the joint hardness distribution is “V-shaped”. The phenomenon is primarily due to the dissolution of strengthening phases in the weld nugget zone, resulting in a hardness lower than that of the base. This alloy, as a high rare earth Mg-RE-Zn alloy, contains a significant amount of the LPSO phase with high melting points. During welding, a considerable portion of the LPSO phase is fragmented into particles and retained, providing a dispersion-strengthening effect. Moreover, dense lamellar structures precipitate within the grains under higher cooling rates. The plane dislocation slip is inhibited effectively and the joint is strengthened. Adding a substantial amount of rare earth elements also hinders grain boundary migration, suppresses grain growth and prevents the formation of weakened regions.

**Figure 9:**
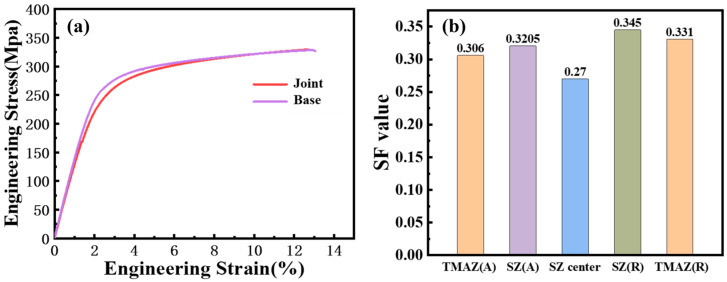
(**a**) Transverse tensile stress–strain curve for optimal parameters; (**b**) base slip Schmidt factor for each zone of the joint under transverse tension.

The transverse tensile strength, plasticity and yield strength of the joint are compared with that of the base. As shown in Figure 9a, the transverse tensile strength of the FSW joint is 329 MPa, which is essentially equivalent to the tensile strength of the base. The welding coefficient of the welded joint exceeds 95%. However, the plasticity and yield strength are slightly lower than that of the base. The lower yield strength than the base is primarily associated with the welding texture [34,35]. The Schmitt factor of the base plane slip in each region of the joint is shown in Figure 9b. When the joint is tensioned transversely, the edge region of the weld nugget zone possesses a larger Schmid factor for the basal plane slip. Some grains exhibit a soft orientation and deform prematurely. This will lead to a premature yield of the material. The inferior plasticity of the joint compared with the base is mainly due to the significant differences in the plastic deformation capacity of various micro-regions. The hardness of the weld nugget zone is greater than that of the base, making it difficult to deform, which results in a reduction of overall strain. However, previous studies investigated the plasticity of the joint through transverse tension tests. In reality, as a heterogeneous structure, the transverse tension tests across regions can no longer accurately reflect the elongation rate of the joint. To accurately assess the contribution of different regions of the joint to the elongation rate, this study employs digital image correlation (DIC) technology to observe the real-time train distribution of the joint during the transverse tension process.

**Figure 10:**
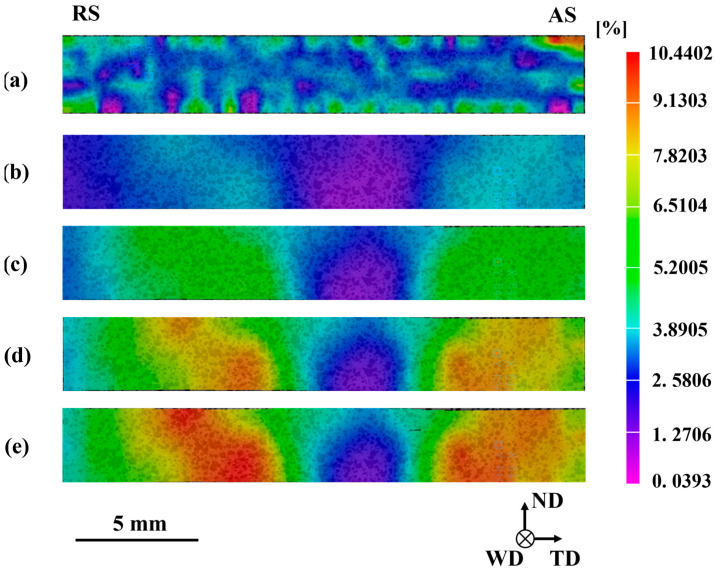
Distribution of extensional strains on the cross-section of the MVWZ842 weld at a global strain of (**a**) 1%; (**b**) 3%; (**c**) 6%; (**d**) 9%; (**e**) 12% (just before fracture).

As shown in Figure 10, the strain cloud map of the joint exhibit a “bowl-shaped” pattern, which is similar to the microstructure morphology. The stir zone with the highest hardness experiences almost zero strain during tensile deformation. The base continuously deforms from the beginning to fracture, with local strains exceeding 10% at the highest. Then, the final fracture location is also on the RS of the base. As shown in Figure 10a,b, the crown zone hardly deforms when the overall deformation is relatively low. The strain is mainly concentrated in the base area at the edge of the TMAZ. The crown zone undergoes about 3% strain when the overall strain is relatively high. The crown zone is wide, the structure is evenly distributed and the contact interface with the base is large. The interface is roughly parallel to the drawing direction. The appropriate deformation of the crown zone can effectively alleviate the stress concentration at the center of the weld. It is worth noting that a significant strength difference exists between the base and the weld nugget zone. Due to the existence of transition zones such as the TMAZ, the strain does not concentrate directly at the interface of the weld nugget zone throughout the entire tensile process. The continuous deformation of the transition zone results in a continuous gradient change in strain between the base and the weld nugget zone. The deformation mismatch between the weld nugget zone and the base is mitigated effectively.

## 4. Discussion

### 4.1. The Mechanisms of Grain Refinement

**Figure 11:**
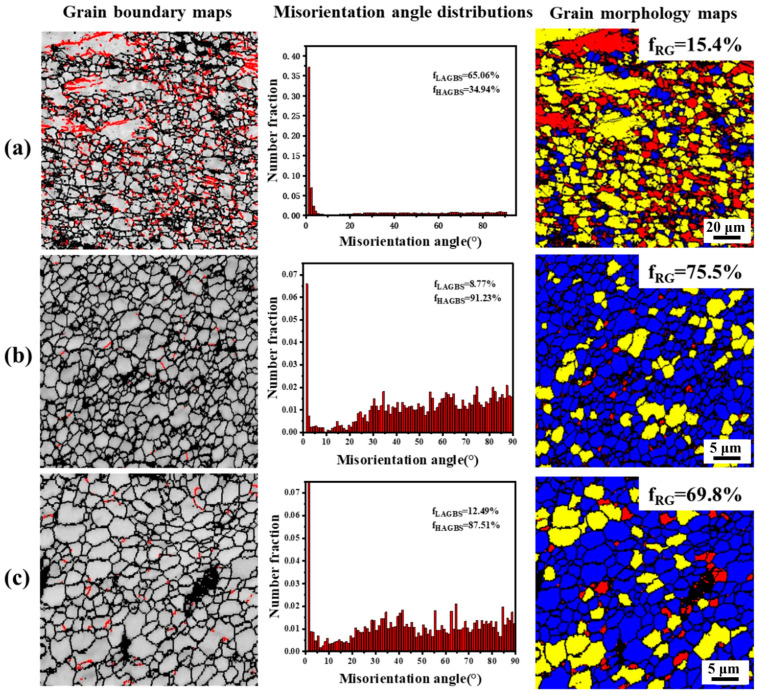
Grain boundary maps, misorientation angle distributions and grain morphology maps of each joint area (the left and right sides are the same area). (**a**) Base; (**b**) nugget zone; (**c**) transition zone.

The grain boundaries, orientation difference angle distribution and recrystallization degree in each region of the joint are shown in Figure 11. In the grain boundary maps, the red line indicates the low-angle grain boundary (2° to 10°). The black line indicates the high-angle grain boundary (greater than 10°). In the recrystallization degree chart, blue represents recrystallized grains, yellow represents subcrystalline grains and red represents deformed grains. As shown in Figure 11, the base contains many low-angle grain boundaries, with a percentage of 65.06%. The recrystallization degree is only 15.4%, and the grains are composed of subcrystalline and deformed grains. This indicates that the temperature of the base in the hot extrusion process is low and the recrystallization is insufficient. However, a very small amount of low-angle grain boundary is distributed in the weld nugget zone, and the percentage is only 8.77%. The grains are mainly composed of recrystallized grains (75.5%) and a small amount of subcrystals. It can be seen that, under high temperatures and severe deformation, the weld nugget undergoes a relatively complete DRX. Low-angle grain boundary number (LAGBS) indicates a small degree of recrystallization. Because FSW cannot provide continuous high temperatures, some low-angle grain boundaries cannot absorb enough dislocation to convert to high-angle grain boundaries and remain. It can be seen from Figure 11c that the proportion of low-angle grain boundaries in the transition zone is slightly higher than that in the weld nugget zone. The degree of recrystallization is slightly lower than that in the weld nugget zone. This shows that the transition zone is also subjected to strong thermal coupling and the structure transitions continuously.

**Figure 12:**
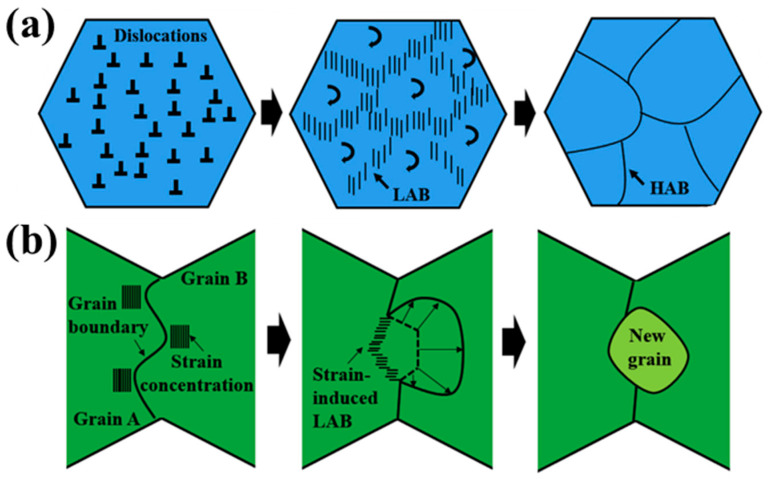
Schematic diagram of grain refinement mechanism. (**a**) CDRX; (**b**) DDRX [37].

The two main mechanisms of dynamic recrystallization are shown in Figure 12. Continuous dynamic recrystallization (CDRX) usually occurs under large deformation and is mainly characterized by grain subdivision [36]. Rare earth magnesium alloy possesses low-level stacking fault energy, which is not easy for dynamic recovery during thermal deformation. It is difficult to offset dislocation by climbing. Instead, dislocation cells are formed by continuous slip and accumulation. Then, dislocation cell polygons form subcrystals. Subcrystals are further absorbed into the dislocation, and the orientation difference of adjacent grains increases into high-angle grain boundaries, forming recrystallized grains. Discontinuous dynamic recrystallization (DDRX) occurs when the strain is small, and its main feature is that the grain boundary protrusion is serrated. When the deformation is not uniform under low strain, a part of the grain boundary of the adjacent grains will arch out into the grains with high-density dislocation. The recrystallization nucleation will occur at this time. Some small-angle grain boundary clusters in the welding nugget zone suggest the existence of discontinuous dynamic recrystallization. Therefore, there are two grain refining mechanisms, CDRX and DDRX, in which CDRX is dominant. With the increased distance from the welding nugget zone, the DDRX mechanism gradually becomes the main grain refining mechanism.

### 4.2. The Mechanisms of Joint Hardening

Due to the heterogeneous structure of the joint, the transverse tensile strength cannot fully represent the performance of the weld nugget zone. There is a three-fold linear relationship between material hardness and yield strength [38], which can accurately reflect the strength properties of the micro-region. Therefore, this section discusses the hardening mechanism of the weld nugget zone based on the previous microstructure analysis and hardness cloud map. 

The hardening mechanism of the welding nugget zone is mainly related to second phase strengthening ∆HV_Orowan_, grain boundary strengthening ∆HV_GB_ and solution strengthening ∆HV_Solution_, namely:

(1) $$\mathrm{HV}={\mathrm{HV}}_{0}+\Delta {\mathrm{HV}}_{\mathrm{GB}}+\Delta {\mathrm{HV}}_{\mathrm{Orowan}}+\Delta {\mathrm{HV}}_{\mathrm{Solution}}+\Delta {\mathrm{HV}}_{\mathrm{other}}$$

In Equation (1), HV_0_ is the hardness of pure magnesium in the annealed state, which is approximately equal to 6 HV [39]. For grain boundary strengthening, the Hall–Petch formula can be used to calculate:

(2) $$∆{\mathrm{HV}}_{GB}=\frac{K_{GB}d^{-\frac{1}{2}}}{3}$$

In Equation (2), K_GB_ is the Holpage constant, the K value of Mg-RE-Zn hot extrusion alloy can be roughly approximated to 164 MPa·μm^−1/2^ and the grain size of the base and the welding nugget zone is obtained by EBSD analysis. Combined with Equation (2), we can obtain $∆{\mathrm{HV}}_{\mathrm{GBBM}}\;$≈ 24.3 HV and $∆{\mathrm{HV}}_{\mathrm{GBSZ}}\;$≈ 45.1 HV.

The second phase strengthening effect can be calculated using Orowan’s Equation [40]:

(3) $${∆HV}_{Orowan}=\frac{Gb}{6\pi \lambda \sqrt{1-v}}ln\left(\frac{d_{t}}{r_{0}}\right)$$

where G is the shear modulus of the magnesium matrix 16.6 GPa, b is the sliding Boehlenberg vector 0.32 nm, v is the Poisson ratio 0.35, d_t_ is the mean particle diameter, r_0_ is the core radius of the dislocation, approximately equal to the Boehlenberg vector b, and λ is the obstacle distance. λ is related to the orientation and shape of the precipitated phase [41]. The LPSO is the base phase, and its phase particles can be assumed to be triangular in distribution on the base surface, so the effective spacing λ is:

(4) $$\lambda =\left(\frac{0.953}{\sqrt{f}}-1\right)d_{t}$$

where f is the volume fraction of the LPSO, and its quantity and size are measured by Image-pro v10 software by combining SEM-BSE and TEM images. The second phase second phase strengthening in the base and the weld nugget zone is $∆{\mathrm{HV}}_{\mathrm{OrowanBM}}\;$≈ 26.3 HV and $∆{\mathrm{HV}}_{\mathrm{OrowanWN}\;}$ ≈ 42.2 HV, respectively. Rare earth elements Gd, Y and Zn have high solid solubility in magnesium. They all have a good solid solution-strengthening effect on the alloy. But, Mn usually exists in the matrix mainly as a single substance. The solid solution strengthening value can be calculated by Equation (5):

(5) $$\Delta {\mathrm{HV}}_{\mathrm{Solution}}=\frac{\sigma_{\mathrm{Solution}}-\sigma_{\mathrm{pure}}}{3}=\frac{1}{3}{\left(\sum_{i}k_{i}^{\frac{1}{n}}C_{i}\right)}^{n}$$

where $\sigma_{Solution}$ is the yield strength of solid solution, $\sigma_{pure}$ is the yield strength of pure magnesium, K_i_ is the strengthening constant of solid solution element i, C_i_ is the concentration of solid solution element i and n is the constant. K_Gd_, K_Y_ and K_Zn_ are 683 MPa·at%^−1/2^, 737 MPa·at%^−1/2^ and 578 MPa·at%^−1/2^, respectively. The concentrations of Gd, Y and Zn are obtained by EDS. The solution strengthening effects in the base and the weld nugget zone are $∆\sigma_{\mathrm{solutionBM}}\;$≈ 35.7 HV and $∆\sigma_{\mathrm{solutionSZ}}$≈ 30.8 HV, respectively.

**Figure 13:**
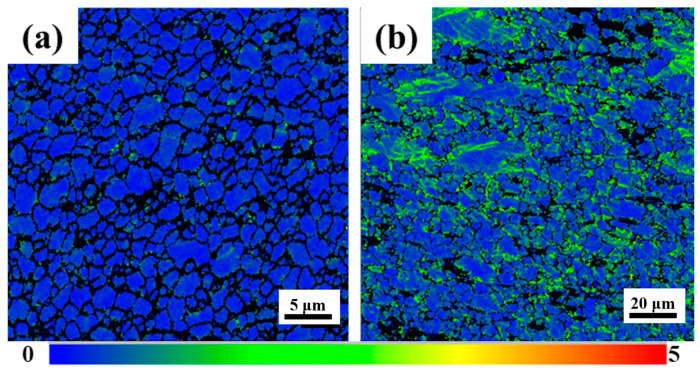
Local area misorientation map (from 0 to 5 represents an increase in the degree of dislocation density). (**a**) Nugget zone; (**b**) base.

**Figure 14:**
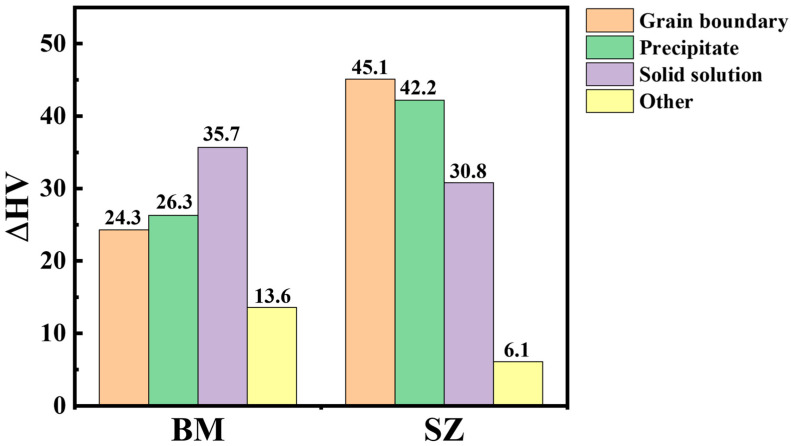
Contribution of each mechanism to the hardness of base and weld nugget zone.

Figure 13 shows the local orientation difference between the weld nugget zone and the base, which can qualitatively reflect the dislocation density. As shown in Figure 13a, the recrystallization of the weld nugget zone is complete. Most of the grains contain very low dislocation density, and their contribution to dislocation hardening is negligible. As can be seen from Figure 13b, due to insufficient heat during extrusion, the base does not fully return to recrystallization. A large number of dislocations remain in the grain, which has a strong work hardening effect. The hardness of the base is about 100 HV, and the weld nugget zone is about 123 HV. The contribution of each strengthening mechanism to the hardness of the MVWZ842 alloy base and the weld nugget zone is shown in Figure 14. It can be seen that the main strengthening mechanisms of the weld nugget zone are grain boundary strengthening and second phase strengthening.

## 5. Conclusions

In this work, the microstructure and mechanical properties of FSW joints of the MVWZ842 alloy are studied, and the hardening mechanism of the joint is discussed in combination with the microstructure. The conclusions are as follows:

(1) The MVWZ842 alloy achieves high-quality connection after friction stir welding, the joint has no holes and cracks and the materials on both sides are fully mixed, with obvious onion ring characteristics.
(2) The grain in the weld nugget zone is obviously refined. The LPSO at the interface of AS and RS is deformed and twisted, and there is an obvious transition of structure. The second phase in the weld nugget zone is not completely dissolved but is significantly broken and dispersed on the matrix. A large number of nanometer α-Mn elements are also distributed in the matrix, which has the effect of refining grains. In addition, stacking fault structures are precipitated in the grain.
(3) Complete dynamic recrystallization occurs in the weld nugget zone, and the recrystallization fraction reaches 75%. The welding texture is formed in the weld nugget zone under the action of rotating shear, but the texture orientation is relatively discrete due to the large presence of Gd and Y elements.
(4) The hardness of the micro-surface shows that the hardness of the weld nugget zone is significantly higher than that of the base, and the highest hardness is 123 HV on the AS of the stir zone. The hardness of all areas of the joint is not lower than that of the base, indicating that there is no area of performance weakening caused by welding.
(5) There is no strain in the stir zone during the transverse tension, and the strain is mainly concentrated in the base at the edge of the thermo-mechanical affected zone. The existence of the transition zone makes the strain not concentrate directly on the edge of the weld nugget zone but change gradiently along the transition zone.
(6) The strengthening mechanism of the joint is mainly fine crystal strengthening, second phase strengthening and solid solution strengthening. Through quantitative calculation, it is found that fine crystal strengthening and second phase strengthening contribute most to the strength.

However, in practical engineering applications, in addition to weld strength, residual stress, anti-corrosion performance and fatigue performance of the joint also need to be considered. The next step is to conduct research on various properties of the joint. In addition, the joint also has a good time hardening effect. It also can be attempted to modify MVWZ842 alloy by friction stir machining to study the feasibility of preparing its high-strength sheet.

## Figures and Tables

**Table 1:** Actual chemical composition of MVWZ842 alloy.

	Mg	Gd	Y	Zn	Mn
MVWZ842	Bal.	8.2167	3.9995	1.2922	0.9623

**Table 2:** Mechanical properties of MVWZ842 alloy.

Alloy	Tensile direction	Hardness (HV)	UTS (MPa)	YS (MPa)	EL (%)
MVWZ842	⊥ED	95.7	328.4	229.5	10.7

**Table 3:** EDS pointing analysis for the positions in Figure 3.

Position	Gd (at.%)	Y (at.%)	Zn (at.%)	Mn (at.%)	Phase
1	3.2	4.1	5.4	0.6	LPSO
2	2.6	2.8	3.2	0.7	LPSO
3	6.6	17.5	——	——	Mg_5_(RE)
4	3.1	3.8	4.9	0.6	LPSO

## Data Availability

The data presented in this study are available on request from the corresponding author.

## References

[1] Bai J.Y., Yang Y., Wen C., Chen J., Zhou G., Jiang B., Peng X.D., Pan F.S. (2023). Applications of magnesium alloys for aerospace: A review. J. Magnes. Alloys.

[2] Ahmadi M., Tabary S.A.A.B., Rahmatabadi D., Ebrahimi M.S., Abrinia K., Hashemi R. (2022). Review of selective laser melting of magnesium alloys: Advantages, microstructure and mechanical characterizations, defects, challenges, and applications. J. Mater. Res. Technol.-JMR T.

[3] Rahmatabadi D., Tayyebi M., Najafizadeh N., Hashemi R., Rajabi M. (2021). The influence of post-annealing and ultrasonic vibration on the formability of multilayered Al5052/MgAZ31B composite. Mater. Sci. Technol..

[4] Zhang J.H., Liu S.J., Wu R.Z., Hou L.G., Zhang M.L. (2018). Recent developments in high-strength Mg-RE-based alloys: Focusing on Mg-Gd and Mg-Y systems. J. Magnes. Alloys.

[5] Liu H., Ju J., Yang X.W., Yan J.L., Song D., Jiang J.H., Ma A.B. (2017). A two-step dynamic recrystallization induced by LPSO phases and its impact on mechanical property of severe plastic deformation processed Mg_97_Y_2_Zn_1_ alloy. J. Alloys Compd..

[6] Li J.Y., Wang F.L., Zeng J., Zhao C.Y., Jin L., Dong J. (2022). Effect of the interspacing of intragranular lamellar LPSO phase on dynamic recrystallization behaviors of Mg-Gd-Y-Zn-Zr alloys. Mater. Charact..

[7] Zhu Y.M., Morton A.J., Nie J.F. (2010). The 18R and 14H long-period stacking ordered structures in Mg-Y-Zn alloys. Acta Mater..

[8] Yamasaki M., Sasaki M., Nishijima M., Hiraga K., Kawamura Y. (2007). Formation of 14H long period stacking ordered structure and profuse stacking faults in Mg-Zn-Gd alloys during isothermal aging at high temperature. Acta Mater..

[9] You S., Huang Y., Kainer K.U., Hort N. (2017). Recent research and developments on wrought magnesium alloys. J. Magnes. Alloys.

[10] Rao H.M., Rodriguez R.I., Jordon J.B., Barkey M.E., Guo Y.B., Badarinarayan H., Yuan W. (2014). Friction stir spot welding of rare-earth containing ZEK100 magnesium alloy sheets. Mater. Des..

[11] Matsuda M., Ii S., Kawamura Y., Ikuhara Y., Nishida M. (2004). Interaction between long period stacking order phase and deformation twin in rapidly solidified Mg_97_Zn_1_Y_2_ alloy. Mater. Sci. Eng. A-Struct. Mater. Prop. Microstruct. Process..

[12] Homma T., Kunito N., Kamado S. (2009). Fabrication of extraordinary high-strength magnesium alloy by hot extrusion. Scr. Mater..

[13] Huang S., Wang J.F., Hou F., Huang X.H., Pan F.S. (2014). Effect of Gd and Y contents on the microstructural evolution of long period stacking ordered phase and the corresponding mechanical properties in Mg-Gd-Y-Zn-Mn alloys. Mater. Sci. Eng. A-Struct. Mater. Prop. Microstruct. Process..

[14] Liu S.J., Wang K., Wang J.F., Huang S., Gao S.Q., Peng X., Hu H., Pan F.S. (2019). Ageing behavior and mechanisms of strengthening and toughening of ultrahigh-strength Mg-Gd-Y-Zn-Mn alloy. Mater. Sci. Eng. A-Struct. Mater. Prop. Microstruct. Process..

[15] Wang K., Dou X.X., Wang J.F., Huang Y.D., Gavras S., Hort N., Liu S.J., Hu H., Wang J.X., Pan F.S. (2020). Achieving enhanced mechanical properties in Mg-Gd-Y-Zn-Mn alloy by altering dynamic recrystallization behavior via pre-ageing treatment. Mater. Sci. Eng. A-Struct. Mater. Prop. Microstruct. Process..

[16] Du W., Wu Y., Nie Z., Su X., Zuo T. (2006). Effects of rare earth and alkaline earth on magnesium alloys and their applications status. Rare Met. Mater. Eng..

[17] Pan H., Ren Y., Fu H., Zhao H., Wang L., Meng X., Qin G. (2016). Recent developments in rare-earth free wrought magnesium alloys having high strength: A review. J. Alloys Compd..

[18] Zuo D., Ding H., Zhi M., Xu Y., Zhang Z., Zhang M. (2024). Research Progress on the Oxidation Behavior of Ignition-Proof Magnesium Alloy and Its Effect on Flame Retardancy with Multi-Element Rare Earth Additions: A Review. Materials.

[19] Liu B.-Y., Liu F., Yang N., Zhai X.-B., Zhang L., Yang Y., Li B., Li J., Ma E., Nie J.-F. (2019). Large plasticity in magnesium mediated by pyramidal dislocations. Science.

[20] Mishra R.S., Ma Z.Y. (2005). Friction stir welding and processing. Mater. Sci. Eng. R-Rep..

[21] Ovid’ko I.A., Valiev R.Z., Zhu Y.T. (2018). Review on superior strength and enhanced ductility of metallic nanomaterials. Prog. Mater. Sci..

[22] Guo Z., Ma T., Yang X., Tao J., Li J., Li W., Vairis A. (2023). In-situ investigation on dislocation slip concentrated fracture mechanism of linear friction welded dissimilar Ti17(α+β)/Ti17(β) titanium alloy joint. Mater. Sci. Eng. A.

[23] Cunha P.H.C.P.d., Lemos G.V.B., Bergmann L., Reguly A., Santos J.F.d., Marinho R.R., Paes M.T.P. (2019). Effect of welding speed on friction stir welds of GL E36 shipbuilding steel. J. Mater. Res. Technol..

[24] Heidarzadeh A., Mironov S., Kaibyshev R., Cam G., Simar A., Gerlich A., Khodabakhshi F., Mostafaei A., Field D.P., Robson J.D. (2021). Friction stir welding/processing of metals and alloys: A comprehensive review on microstructural evolution. Prog. Mater. Sci..

[25] Mosayebi M., Zarei-Hanzaki A., Tahaghoghi M., Abedi H.R., Moshiri A., Ghaderi A. (2022). Effect of second phase particles on the microstructure and texture of rare earth elements containing magnesium matrix surface-composite produced by friction stir processing. J. Mater. Res. Technol.-JMR T.

[26] Sivashanmugam N., Harikrishna K.L., Rao S.R.K., Justin S.J.S., Wilson P. (2023). Mechanical and corrosion characteristics of micro-arc oxidized magnesium alloy (ZE41) friction stir welds in modified SBF. Phys. Scr..

[27] da Silva E.P., Oliveira V.B., Pereira V.F., Maluf O., Buzolin R.H., Pinto H.C. (2017). Microstructure and Residual Stresses in a Friction Stir Welded Butt Joint of as-cast ZK60 Alloy Containing Rare Earths. Mater. Res.-Ibero-Am. J. Mater..

[28] Krishnan K.N. (2002). On the formation of onion rings in friction stir welds. Mater. Sci. Eng. A-Struct. Mater. Prop. Microstruct. Process..

[29] Xin R., Zheng X., Liu Z., Liu D., Qiu R., Li Z., Liu Q. (2016). Microstructure and texture evolution of an Mg-Gd-Y-Nd-Zr alloy during friction stir processing. J. Alloys Compd..

[30] Liang M.C., Zhang H., Zhang L.F., Xue P., Ni D.R., Wang W.Z., Ma Z.Y., Ye H.Q., Yang Z.Q. (2021). Evolution of Quasicrystals and Long-Period Stacking Ordered Structures During Severe Plastic Deformation and Mixing of Dissimilar Mg Alloys Upon Friction Stir Welding. Acta Metall. Sin.-Engl. Lett..

[31] Yang Q., Xiao B.L., Wang D., Zheng M.Y., Ma Z.Y. (2015). Study on distribution of long-period stacking ordered phase in Mg-Gd-Y-Zn-Zr alloy using friction stir processing. Mater. Sci. Eng. A-Struct. Mater. Prop. Microstruct. Process..

[32] Jiang M.G., Xu C., Yan H., Nakata T., Chen Z.W., Lao C.S., Chen R.S., Kamado S., Han E.H. (2021). Quasi-in-situ observing the rare earth texture evolution in an extruded Mg-Zn-Gd alloy with bimodal microstructure. J. Magnes. Alloys.

[33] Liu D.J., Xin R.L., Sun L.Y., Zhou Z., Liu Q. (2013). Influence of sampling design on tensile properties and fracture behavior of friction stir welded magnesium alloys. Mater. Sci. Eng. A-Struct. Mater. Prop. Microstruct. Process..

[34] Xin R.L., Li B., Liao A.L., Zhou Z., Liu Q. (2012). Correlation Between Texture Variation and Transverse Tensile Behavior of Friction-Stir-Processed AZ31 Mg Alloy. Metall. Mater. Trans. A-Phys. Metall. Mater. Sci..

[35] Wang Y.N., Chang C.I., Lee C.J., Lin H.K., Huang J.C. (2006). Texture and weak grain size dependence in friction stir processed Mg-Al-Zn alloy. Scr. Mater..

[36] Lin P.T., Liu H.C., Hsieh P.Y., Wei C.Y., Tsai C.W., Sato Y.S., Chen S.C., Yen H.W., Lu N.H., Chen C.H. (2021). Heterogeneous structure-induced strength-ductility synergy by partial recrystallization during friction stir welding of a high-entropy alloy. Mater. Des..

[37] Xu N., Song Q.N., Bao Y.F., Fujii H. (2019). Investigation on microstructure and mechanical properties of cold source assistant friction stir processed AZ31B magnesium alloy. Mater. Sci. Eng. A-Struct. Mater. Prop. Microstruct. Process..

[38] Zhang P., Li S.X., Zhang Z.F. (2011). General relationship between strength and hardness. Mater. Sci. Eng. A-Struct. Mater. Prop. Microstruct. Process..

[39] Sun W.T., Qiao X.G., Zheng M.Y., Xu C., Kamado S., Zhao X.J., Chen H.W., Gao N., Starink M.J. (2018). Altered ageing behaviour of a nanostructured Mg-8.2Gd-3.8Y-1.0Zn-0.4Zr alloy processed by high pressure torsion. Acta Mater..

[40] Nie J.F. (2012). Precipitation and Hardening in Magnesium Alloys. Metall. Mater. Trans. A-Phys. Metall. Mater. Sci..

[41] Nie J.F. (2003). Effects of precipitate shape and orientation on dispersion strengthening in magnesium alloys. Scr. Mater..

